# A germline alteration of ERBB2 increases the risk of breast cancer in Chinese Han women with a familial history of malignant tumors

**DOI:** 10.3892/ol.2019.10930

**Published:** 2019-09-27

**Authors:** Yan Ju, Lifeng Wang, Shengjun Ta, Rui Shu, Shanling Yang, Xican Gao, Hongping Song, Liwen Liu

Oncol Lett 18: 2885-2890, 2019; DOI: 10.3892/ol.2019.10646

Subsequent to the publication of this article, the authors have realized that the name of the last-listed corresponding author, Liwen Liu, was spelt incorrectly as “Liwen Lu” in the correspondence details, even though the name was printed correctly as Liwen Liu in the author affiliations. Therefore, the correspondence information for this paper should have appeared as follows:

Correspondence to: Dr Hongping Song or Dr Liwen Liu, Department of Ultrasonic Medicine, Xijing Hospital, The Fourth Military Medical University, 172 Chang Le Road, Xi'an, Shaanxi 710032, P.R. China

E-mail: song.hp@foxmail.com

E-mail: liuliwen@fmmu.edu.cn

The authors regret that this this error was not noticed and corrected prior to the publication of the article, and apologize to the readership for any inconvenience caused.

